# The role of the orbitofrontal cortex and the nucleus accumbens for craving in alcohol use disorder

**DOI:** 10.1038/s41398-021-01384-w

**Published:** 2021-05-04

**Authors:** Tobias Bracht, Leila Soravia, Franz Moggi, Maria Stein, Matthias Grieder, Andrea Federspiel, Raphaela Tschümperlin, Hallie M. Batschelet, Roland Wiest, Niklaus Denier

**Affiliations:** 1 University Hospital of Psychiatry and Psychotherapy, University of Bern, Bern, Switzerland; 2 Translational Research Center, University Hospital of Psychiatry and Psychotherapy, University of Bern, Bern, Switzerland; 3 Clinic Suedhang, Kirchlindach, Switzerland; 4 Department of Clinical Psychology and Psychotherapy, Institute of Psychology, University of Bern, Bern, Switzerland; 5 Institute of Diagnostic and Interventional Neuroradiology, University of Bern, Bern, Switzerland

**Keywords:** Addiction, Molecular neuroscience

## Abstract

This study aimed to investigate structural and functional alterations of the reward system and the neurobiology of craving in alcohol use disorder (AUD). We hypothesized reduced volume of the nucleus accumbens (NAcc), reduced structural connectivity of the segment of the supero-lateral medial forebrain bundle connecting the orbitofrontal cortex (OFC) with the NAcc (OFC-NAcc), and reduced resting-state OFC-NAcc functional connectivity (FC). Furthermore, we hypothesized that craving is related to an increase of OFC-NAcc FC. Thirty-nine recently abstinent patients with AUD and 18 healthy controls (HC) underwent structural (T1w-MP2RAGE, diffusion-weighted imaging (DWI)) and functional (resting-state fMRI) MRI-scans. Gray matter volume of the NAcc, white matter microstructure (fractional anisotropy (FA)) and macrostructure (tract length) of the OFC-NAcc connection and OFC-NAcc FC were compared between AUD and HC using a mixed model MANCOVA controlling for age and gender. Craving was assessed using the thoughts subscale of the obsessive-compulsive drinking scale (OCDS) scale and was correlated with OFC-NAcc FC. There was a significant main effect of group. Results were driven by a volume reduction of bilateral NAcc, reduced FA in the left hemisphere, and reduced tract length of bilateral OFC-NAcc connections in AUD patients. OFC-NAcc FC did not differ between groups. Craving was associated with increased bilateral OFC-NAcc FC. In conclusion, reduced volume of the NAcc and reduced FA and tract length of the OFC-NAcc network suggest structural alterations of the reward network in AUD. Increased OFC-NAcc FC is associated with craving in AUD, and may contribute to situational alcohol-seeking behavior in AUD.

## Introduction

Severe alcohol use disorder (AUD) is a chronic relapsing disorder associated with harmful somatic, psychological, and social consequences^1^. Core features of AUD are craving, the recurring desire and urge for alcohol consumption and the impaired ability to control alcohol-seeking behavior despite its negative consequences^2,3^. According to the World Health Organization (WHO) more than 3 million deaths worldwide are caused by harmful use of alcohol each year^4^. AUD is characterized by very high rates of post-residential treatment relapse (80–92%)^5,6^, especially in the first 3 months after residential treatment^7,8^. Thus, there is a need for more effective treatment regimens. A profound knowledge on the neurobiology of addiction, particularly regarding craving and inhibition of alcohol seeking behavior, may contribute to improving novel treatment approaches such as alcohol-specific inhibition training, neurofeedback, or brain stimulation therapies^9–11^. This, in turn may set the path for a more personalized AUD treatment.

On a neurobiological level the reward system plays an essential role for the pathophysiology of addiction^2^, in particular regarding the hedonic experience of consuming alcohol (e.g., liking) and the urge for alcohol consumption (e.g., wanting)^12^. Central relay stations of the reward system are the ventral tegmental area (VTA), the nucleus accumbens (NAcc), and the prefrontal cortex (PFC) including the orbitofrontal cortex (OFC), a core region for the selection of motivationally relevant events^2,3,13^. In healthy participants including heavy social drinkers, liking is moderately associated with wanting, although it has been suggested that wanting and liking are dividable and independent processes^14^. During the progression of addiction there is a decrease of liking and an increase of wanting, the latter being related to the experience of craving and to compulsive alcohol use^15^. Wanting relies on dopaminergic pathways projecting from the VTA to the NAcc and to the OFC^12^. These dopaminergic projections initiate neuroplastic changes in their target regions (e.g., the OFC), which encode for learned associations with pleasurable events (e.g., alcohol consumption)^16^. Such associations may form the basis for situational alcohol-seeking behavior in AUD. Consequently, models of addiction refer to the OFC-NAcc connection as the final common pathway for initiating alcohol-seeking behavior in AUD^2,3^.

Gray matter volume reductions of core regions of the reward system in AUD have been reported repeatedly (e.g., NAcc, OFC^17,18^). This may be due to a particularly pronounced vulnerability of regions of the reward system to the neurotoxic effects of alcohol^19,20^. Structurally, those regions of the reward system are connected through white matter pathways forming a complex network^13^. The most commonly applied measure for the assessment of white matter microstructure is the fractional anisotropy (FA), reflecting the coherence of diffusion of water molecules^21^. A series of voxel-by-voxel comparison-based whole brain studies found FA reductions in AUD^22,23^. Localizations of findings are widespread (e.g., in the corpus callosum, internal and external capsule, or in the fornix) and many findings lack associations with behavioral measures relevant to addiction. Thus, those alterations may rather be the result of alcohol toxicity than a neurobiological correlate of addiction pathophysiology. This assumption is also supported by observations that global white matter microstructure alterations in AUD are reversible, if abstinence is maintained and recur after relapse^24^.

The fiber architecture of the reward system^13^, believed to underlie the pathophysiology of addiction^2,3^, is complex because of multiple crossing and merging fibers^25^. In such regions of crossing fibers, tract-specific conclusions require the use of tractography, allowing for a non-invasive in vivo reconstruction of neuronal pathways based on diffusion weighted MRI^26,27^. However, tractography studies of the reward system in AUD are scarce^28,29^. One previous study used tractography to investigate the supero-lateral branch of the medial forebrain bundle (slMFB) in AUD^28^. The slMFB consists of mono- and polysynaptic projection pathways of the VTA (vtaPP^25,30^,) connecting the VTA with the NAcc and with prefrontal brain regions including the OFC^31^. Based on lesion studies in humans and on tract-tracing studies, such projection pathways likely consist of bidirectional corticopetal and corticofugal projection pathways into and out of the VTA^25,32^. In our study, we investigate the slMFB segment anterior of the NAcc, which predominantly connects to the OFC^30,33,34^. In the following, we refer to this segment of the slMFB as the OFC-NAcc connection. In addition to FA, a measure of white matter microstructure^27^, we use the tract length as a measure of white matter macrostructure, which may be related to white matter atrophy^35^ complementing our structural white matter analyses.

Functional MRI (fMRI) allows for the measurement of fluctuations in blood oxygen level-dependent (BOLD) signals that are associated with specific brain functions. This complements structural analyses of gray matter volume and white matter microstructure. In AUD, there are decreased BOLD-levels of core regions of the reward system in the absence of alcoholic stimuli^3,18,22,36–38^. Extending classical BOLD analyses, resting-state functional connectivity (FC) analyses provide crucial information on connectivity strengths between spatially segregated brain regions, hereby yielding functional networks^39^. Decreased resting-state FC between core regions of the reward system including the NAcc and the OFC^37,38,40^ may be associated with a lack of response to natural rewarding stimuli and result in a psychological state characterized by feelings of anxiety, reduced energy, and restlessness^2,41^.

Consistent with the assumption that patients with severe AUD drink alcohol to diminish aversive affects^42^, there is an enhanced cue reactivity in AUD in response to alcoholic cues in regions of the reward system^18,22,36,43,44^. The OFC and the NAcc seem to be key to the neurobiology of AUD given that both regions have been related to craving in response to alcoholic cues^44–46^. In this study, we extend these previous fMRI analyses linking craving at rest to resting-state OFC-NAcc connectivity, hereby providing information on the neurobiology of craving on a functional network level.

It was the aim of this first three-fold multimodal MRI-study using gray matter volumetry, tractography, and FC analyses to investigate structural and functional aspects of the OFC-NAcc network, and its association with craving in a group of recently abstinent patients with severe AUD compared to healthy controls. Our first hypothesis was decreased bilateral volume of the NAcc in AUD^17,18^. Second, we hypothesized decreased FA and a reduced tract length of the OFC-NAcc connection in AUD^22^. Third, we hypothesized decreased OFC-NAcc resting-state FC in AUD^37,38,40^ and fourth, we hypothesized that increased resting-state OFC-NAcc FC is associated with craving in AUD^44,46^.

## Methods

### Study participants

All AUD-patients were participating in a clinical trial investigating the effects of an alcohol-inhibition training on relapse^47^. A sub-sample of those AUD-patients was included in a longitudinal fMRI-study investigating the functional correlates of this training. The diffusion-weighted MRI-protocol is an extension of the latter study and was added at a later stage. In our study, only baseline MRI-scans and assessments that were acquired before the intervention started were considered. To avoid influences of age-related atrophy on brain structure inclusion criterion for all participants was age between 18–59 years of age. Thirty-nine right-handed detoxified patients with severe AUD attending a 12-week abstinence-oriented residential treatment program for AUD in a specialized treatment center in Switzerland (Clinic Suedhang) were recruited. All patients met criteria for AUD as clinically assessed in accordance with the DSM-5, and had been abstinent for at least 4 weeks prior to study participation. Eighteen right-handed healthy controls (HC) with non-problematic drinking behavior (Alcohol Use Disorders Identification Test (AUDIT^48^) score < 8; Alcohol Use Disorder Scale (AUD-S^49^ score < 2)), and low scores regarding psychopathology (Brief Symptom Check List (BSCL^50^, GSI_*t*-value_ ≤ 63) were recruited. Current treatment for a psychiatric diagnosis and/or psychopharmacological medication, treatment for any substance use disorder in the past, problematic substance use (except nicotine; Drug Use Disorders Identification Test (DUDIT) score ≤ 8 per substance^51^, e.g., cannabis) and neurocognitive problems were exclusion criteria.

This study aimed to investigate the neurobiology of subjective craving at rest (as assessed with resting-state fMRI). Craving was assessed immediately before the MRI-scan and measured with the German version of the obsessive compulsive drinking scale (OCDS)^52^. The OCDS consists of 14 items assessing both obsessive thoughts about alcohol use and compulsive drinking behavior. Given that resting-state fMRI is measured in the absence of behavior by definition, we chose the obsessive thoughts of alcohol subscale as our outcome measure for craving. Furthermore, we used the Comprehensive Alcohol Expectancy Questionnaire (CAEQ), a self-report measure with 41 items, designed to assess an individual’s positive and negative alcohol outcome expectancies, which are related to the level of alcohol use and relapse following treatment for alcohol use disorders^53^. In addition, the brief symptom checklist (global severity index, GSI) was used for the assessment of subjective impairments due to somatic and mental problems^54^. Depressive symptoms were assessed with the Beck depression inventory (BDI)^55^. All participants provided written informed consent and were reimbursed for participation. The study was approved by the local ethics committee (KEK-number: 2016-00988) and registered at Clinicaltrials.gov (NCT02968537) and the Swiss National Clinical Trials Portal (SNCTP000002043).

### MRI-data acquisition

MRI data were acquired at the University Hospital of Bern, with a 3 Tesla Siemens Magnetom Prisma scanner and with a 64-channel head/neck coil. For high-resolution T1-weighted structural images, we used a bias-field corrected MP2RAGE sequence for improved segmentation (256 Slices, field of view (FOV) = 256 × 256, 256 × 256 matrix, 1 mm^3^ isotropic resolution, repletion time (TR) = 5000 ms, echo time (TE) = 2.98 ms, inversion time *T*1 = 700 ms and *T*2 = 2500 ms). The MP2RAGE sequence generated three image volumes, two gradient echo images (INV1 and INV2) and a T1 weighted image (UNI). Diffusion weighted images (DWI) were acquired using a spin-echo echo-planar sequence with 64 non-collinear directions and *b*-value = 1000 s/mm^2^ (60 Slices, FOV = 269 × 269, 128 × 128 matrix, 2.2 mm^3^ isotropic resolution, TR = 6200 ms, TE = 69 ms). A resting-state fMRI scan (condition: eyes closed) was acquired using whole brain echo planar imaging (EPI) (300 volumes with 60 slices per volume, FOV = 230 × 230, 104 × 104 matrix, 2.2 mm^3^ isotropic resolution, TR = 1300 ms, TE = 37 ms).

### Data analyses

#### Structural MRI pre-processing

**Fig. 1:**
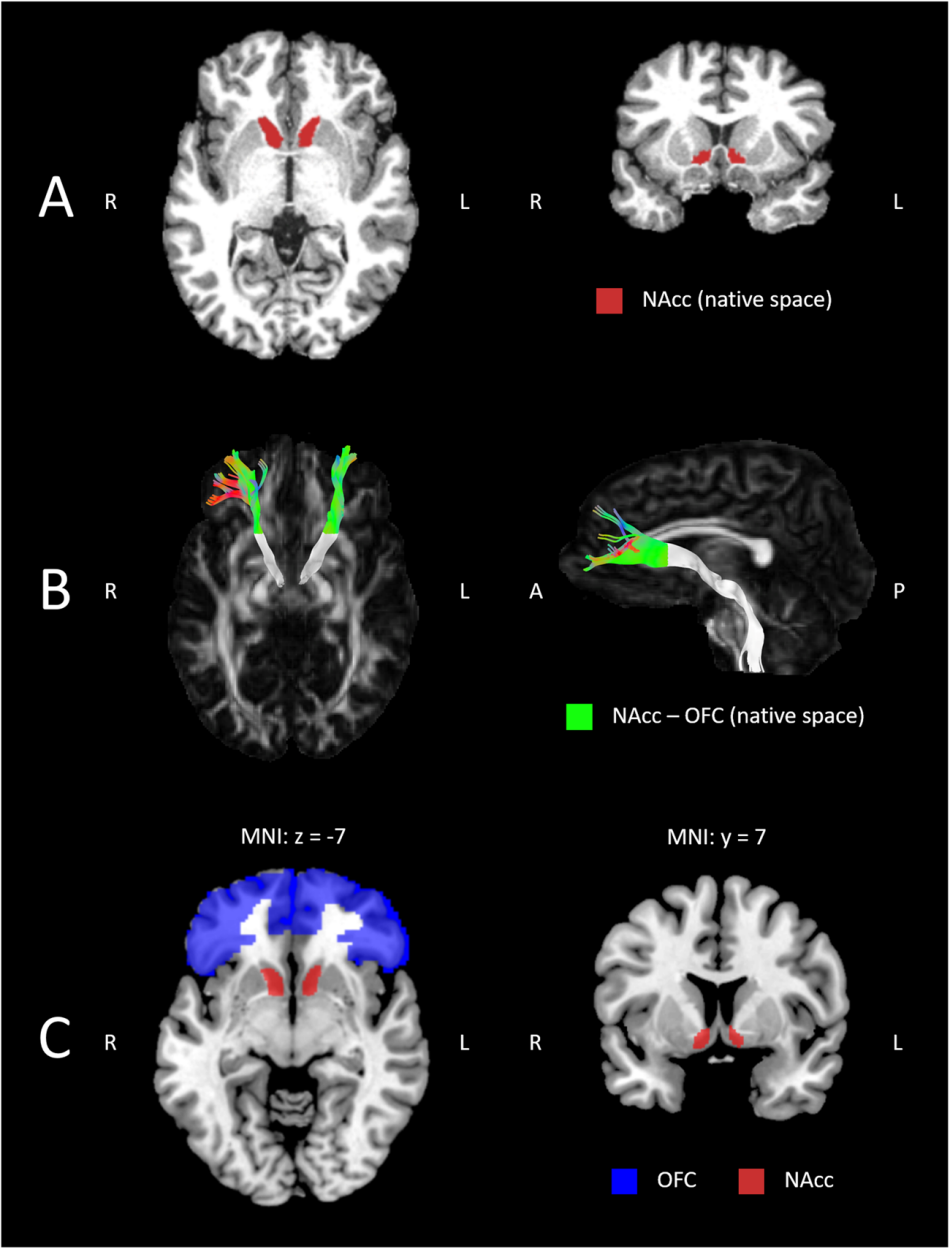
Multimodal imaging analyses. **A** Example volumetry of bilateral NAcc in native space. **B** Example of individual native space OFC-NAcc connections (color coded) and the remaining proximal segment (in gray). **C** OFC (BA 10 and 11) and NAcc masks in MNI space for computation of bilateral OFC-NAcc FC.

We used *FSL-FIRST*, a model-based segmentation and registration tool for subcortical structures implemented in *FSL 5.0* (http://www.fmrib.ox.ac.uk/fsl/first/index.html), for segmentation of bilateral NAcc^56,57^. Due to noisy background of the MP2RAGE UNI image, we used the INV2 image for brain extraction and binary mask generation with *FSL-BET* using robust brain center estimation (-R option). Then, we applied the binary mask to the UNI image using *FSLMATHS* and run *FSL-FIRST* for segmentation of the bilateral NAcc using the brain extracted UNI image as input and standard settings. The segmentation model uses a Bayesian appearance model and measures the probabilistic relationships between shape and GM intensity. Subcortical volumes (in mm^3^) of the bilateral NAcc were finally determined using *FSL-STATS* with the *all_fast_firstseg* files as input and a threshold of 25.5–26.5 for the left NAcc and 57.5–58.5 for the right NAcc (Fig. 1A).

#### Diffusion-weighted MRI data pre-processing

Diffusion-weighted MRI (DW-MRI) data were analyzed using *ExploreDTI 4.8.6*^58^. Data were pre-processed as described in previous publications^59,60^. In short, a subjection motion and distortion correction and an EPI-correction was performed^61^. Whole-brain tractography was executed fitting a single diffusion tensor model to the DW-MRI data^62^. Angle threshold of >45° and FA <0.2 were used as termination criteria. Diffusion properties (e.g., FA) were sampled along the tracts.

#### Tract reconstruction

We aimed at reconstructing the OFC-NAcc segment of the slMFB, because this segment is part of a polysynaptic network connecting OFC, NAcc and VTA^31^, core regions for the pathophysiology of craving in AUD^2,3^. First, the entire slMFB was reconstructed as described in previous publications^34,35^. The following seed gates were used separately for both hemispheres: The VTA was identified on a horizontal section. A circular seed was drawn anterior of the red nucleus, medial of the substantia nigra and posterior to the mammillary bodies. A second seed region was drawn on a coronal section at the height of the NAcc surrounding the anterior limb of the internal capsule. Seed regions were chosen in line with previous studies (e.g.,^34,35^). Given that we were specifically interested in the OFC-NAcc segment of the MFB, we used the splitter tool implemented in *ExploreDTI 4.8.6* to isolate the slMFB segment anterior of the NAcc connecting to (predominantly orbitofrontal) prefrontal brain regions (see Fig. 1B). Mean-FA and tract length were calculated for each tract^35^.

#### Functional MRI pre-processing

We analysed FC using a seed-driven approach of the *CONN 19c* toolbox^63^. We used standard pipeline and parameters. Pre-processing steps included realignment and field map correction of EPI volumes, co-registration to structural volumes (MP2RAGE) and segmentation/normalization of structural volumes and smoothing of normalized EPI volumes. We applied a band-pass filtering (0.008–0.09 Hz) to remove physiological signals and regressed nuisance variables including 12 realignment parameters and each five time series within segmented white matter and cerebrospinal fluid, derived by principal component analysis. Afterwards, we defined the left and right NAcc as seed region and computed their regional FC over the whole brain. Using *MATLAB*, we extracted mean values of FC-maps within the left and right OFC using predefined masks representing Brodmann area 10 and 11 of the *WFUPickatlas 3.0* (see Fig. 1C).

### Statistical analyses

Based previous studies investigating functional^37^ and structural alterations^17,28^ of the reward system in AUD, we expected to get a mean effect size of *f*^2^ = 0.15 for the between factor (AUD vs. HC) having a total sample size of 57 participants and performing a mixed-model MANCOVA with one between factor, one within factor, four dependent variables and controlling for the two covariates age and gender. A posthoc analysis to calculate the power achieved, given alpha = 0.05, sample size = 57, number of groups = 2, number of dependent variables = 4, and number of covariates = 3, yielded a power (1-beta) of 0.93 using the program G*Power^64^.

Statistical analyses were performed using Statistical Package for Social Sciences SPSS 26.0 (SPSS, Inc., Chicago, Illinois). Demographics between AUD und HC were compared using *t*-tests for continuous variables or *χ*^2^ tests for dichotomous variables. A mixed-model MANCOVA controlling for age and gender with the between-factor group (HC vs. AUD), the within-factor hemisphere (left, right) and four dependent variables comprising the four modalities (gray matter volume of NAcc, FA (OFC-NAcc), tract length (OFC-NAcc) and FC (OFC-NAcc)) was used. Significant main effects were followed up with post-hoc tests. In case of a significant group × hemisphere interaction, post-hoc ANCOVAs controlling for age and gender were calculated separately for each hemisphere and modality. All tests were two-tailed and a probability of <0.05 was considered statistically significant. Effect sizes were reported as *η*^2,65^. Additional exploratory correlations for the AUD group were calculated between the four imaging modalities and the total OCDS-scale and the compulsive drinking subscale (see Supplementary material Tables 1 and 2). To provide more detailed anatomical information on the localization of FC, two separate seed-based FC analyses were performed using the NAcc as a seed. First, NAcc-FC to the whole brain and second to the OFC mask. Analyses were performed separately for each hemisphere using the CONN toolbox^63^. Voxel-wise comparisons in FC between AUD and HC groups were performed with a voxel-level threshold of *p* < 0.001 and with a family wise error (FWE) cluster-level correction of *p* < 0.05.

## Results

### Study population

The groups did not differ regarding age or gender. Results indicate that our AUD group mainly consists of patients with severe AUD (see Table 1 for demographics and clinical characteristics).

**Table 1:** Sociodemographic and clinical characteristics.

	AUD patients (*N* = 39)	Healthy controls (*N* = 18)	*p*-values
Females/males	15/24	6/12	0.709
Age	41.92 (8.55)	40.44 (12.12)	0.645
Relationship (yes/no)	20/19	15/3	0.021*
Years of education	14.21 (3.76)	16.33 (3.46)	0.047*
Employment (yes/no)	23/16	17/1	0.007**
Number of detoxifications	2.89 (2.92)		
Days of abstinence	30.32 (14.65)		
Years of problematic drinking	11.25 (9.17)		
AUDIT	24.43 (7.25)	4.00 (1.88)	<0.001***
AUD	29.40 (6.83)	0.83 (1.30)	<0.001***
OCDS-Thoughts	4.59 (3.85)	0.11 (0.32)	<0.001***
CAEG	3.37 (0.46)		
BDI II	15.70 (9.17)		
BSCL GSI	1.21 (0.61)	0.18 (0.19)	<0.001***

Means and standard deviations are displayed. Group differences are displayed by *p < 0.05, **p < 0.01, and ***p < 0.001.

*AUDIT* alcohol use disorders identification test, *AUD* alcohol use disorder scale, *OCDS* obsessive compulsive drinking scale, *CAEG* comprehensive alcohol expectancy questionnaire, *BDI* Beck depression inventory, Brief symptom check list.

### Group comparisons

**Fig. 2:**
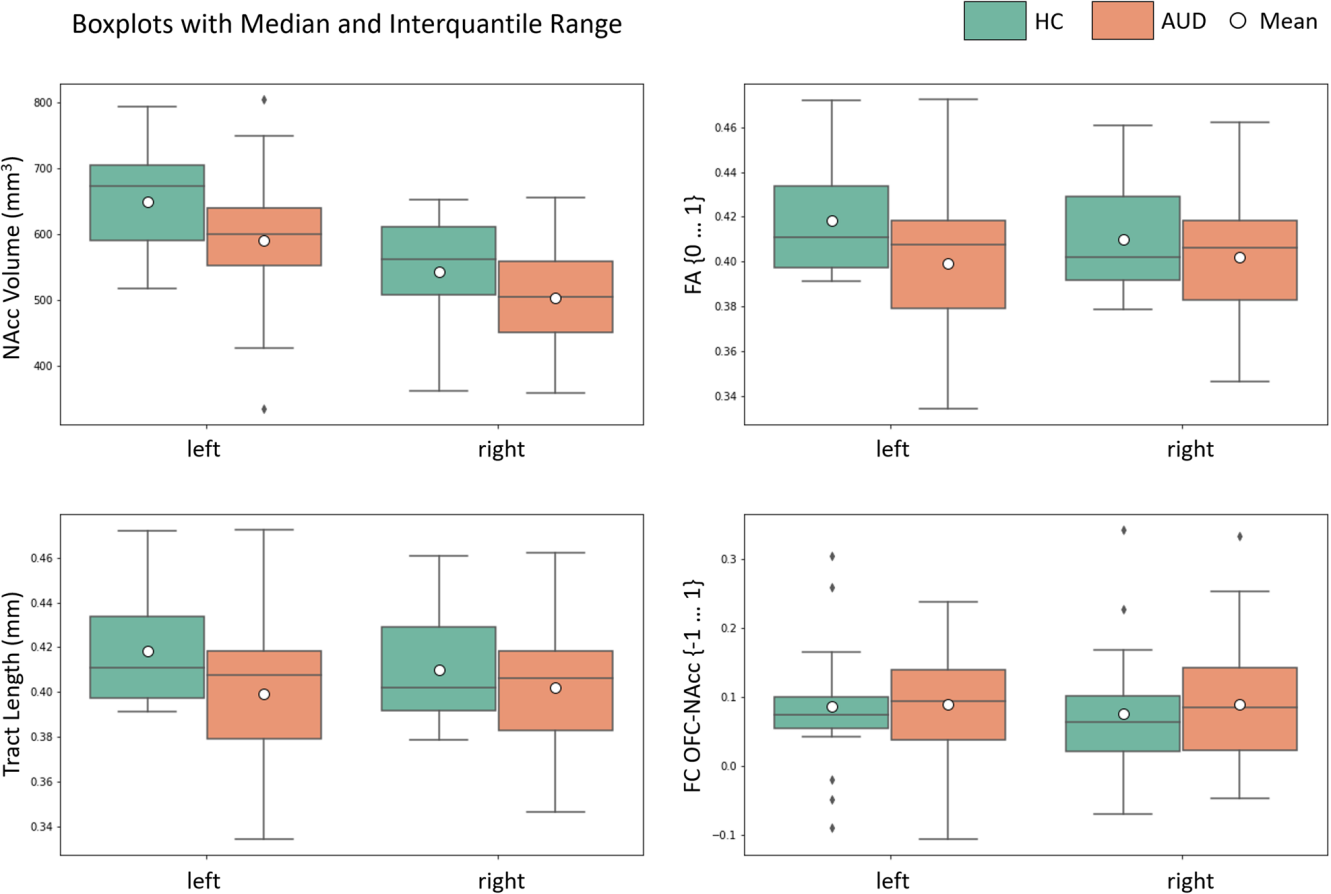
Boxplots for AUD and HC are displayed for the respective imaging modalities. There was a significant group main effect for NAcc volume (bilateral), for FA of the left OFC-NAcc segment and for tract length (bilateral). OFC-NAcc FC did not differ between groups.

The mixed model MANCOVA revealed a significant main effect of group showing a large effect size (*F*(4, 50) = 2.612, *p* = 0.046, *η*^2^ = 0.173). Posthoc tests demonstrated a significant group main effect for NAcc volume (AUD patients < controls; *F*(1, 53) = 5.986, *p* = 0.018, *η*^2^ = 0.101, hemisphere × group interaction (*F*(1, 53) =0.458, *p* = 0.502, *η*^2^ = 0.009). Further posthoc tests revealed a significant hemisphere × group interaction for FA (*F*(1, 53) = 4.629, *p* = 0.036, *η*^2^ = 0.080). Therefore, separate ANCOVAs with the independent variable group and the dependent variable FA controlling for age and gender were calculated for each hemisphere. FA of the OFC-NAcc connection was reduced in AUD in the left (*F*(1, 53) = 5.224, *p* = 0.026, *η*^2^ = 0.090), but not in the right (*F*(1, 53) = 0.803, *p* = 0.374, *η*^2^ = 0.015) hemisphere. Finally, there was a significant main effect for OFC-NAcc tract length (AUD patients < controls; *F*(1, 53) = 4.609, *p* = 0.036, *η*^2^ = 0.080, hemisphere × group interaction (*F*(1, 53) = 0.015, *p* = 0.902, *η*^2^ < 0.001), but not for resting state OFC-NAcc FC (*F*(1, 53) = 0.152, *p* = 0.698, *η*^2^ = 0.003, hemisphere × group interaction (*F*(1, 53) = 0.198, *p* = 0.659, *η*^2^ = 0.004) (see Fig. 2). For both hemispheres, we found no significant group differences between AUD and HC in seed-based FC from the NAcc to the whole brain and to the OFC mask. See supplementary Fig. 1 for visualization of whole-brain FC within group and hemisphere.

### Correlations with craving and OFC-NAcc FC

**Fig. 3:**
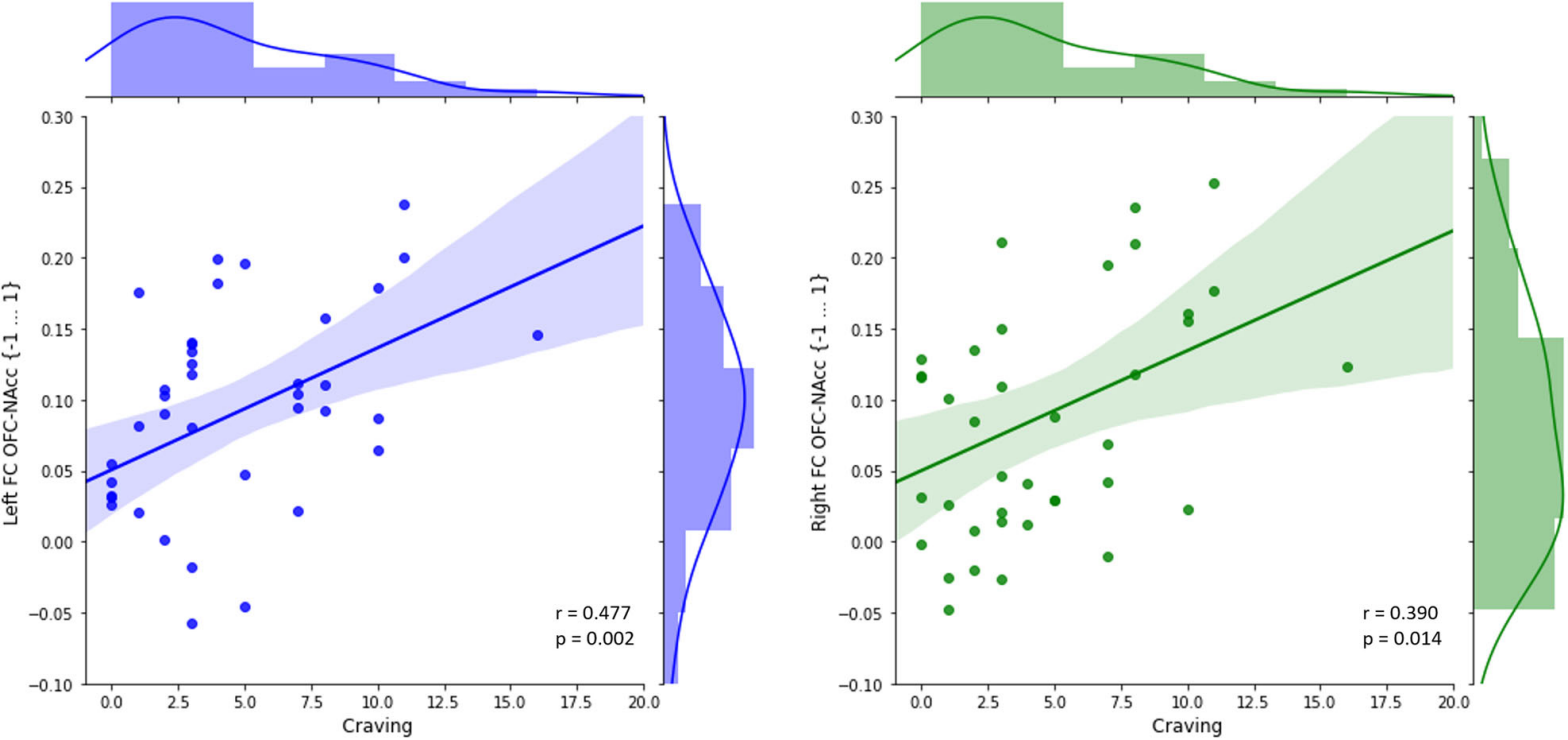
Correlations of OFC-NAcc FC with craving in AUD.

In the AUD group craving was significantly correlated with OFC-NAcc FC for both the left (*r* = 0.477, *p* = 0.002) and the right (*r* = 0.390, *p* = 0.014) hemisphere, indicating higher FC with increased craving (see Fig. 3).

## Discussion

This is the first study that combined gray matter volume analyses of the NAcc, tractography of the OFC-NAcc segment of the slMFB and OFC-NAcc FC to investigate structural and functional alterations in AUD and its association with craving. Our results point to structural alterations of the OFC-NAcc network in recently abstinent patients suffering from severe AUD. We found reduced gray matter volume of the NAcc, and impaired structural connectivity in terms of reduced white matter microstructure (FA) of the left and macrostructure (tract length) of bilateral OFC-NAcc connections. There were no group differences in resting-state OFC-NAcc FC. Bilateral OFC-NAcc resting-state FC positively correlated with craving, thus underpinning this network’s key role for individual variations in the pathophysiology of AUD.

One anatomical concept regarding the OFC-NAcc pathway is that this final common pathway activates the NAcc^2^, which in turn projects to the pallidum initiating alcohol-seeking behavior via thalamo-cortical pathways^3^. An alternative explanation is that OFC-NAcc projections of the slMFB activate corticofugal glutamatergic projections from the NAcc to the VTA (as visualized by the gray shaded segment of the slMFB (Fig. 1B)). Dopaminergic efferents from the VTA may project to the PFC via the infero-medial MFB (the classic anatomical MFB description in rodents)^33,66^, hereby mediating craving. Both theories are consistent with results of exploratory correlations suggesting that higher FA of the left OFC-NAcc is associated with increased craving in AUD (*r* = 0.321, *p* = 0.046, see supplementary material, Table 1). Thus, white matter microstructure of the OFC-NAcc pathway may indeed be associated with craving, indicating a core role of this pathway for the pathophysiology of addiction.

This assumption is indirectly supported by our resting-state fMRI-results. We identified a positive correlation between left and right resting-state OFC-NAcc FC and craving. Consequently, the OFC-NAcc network may play an important role for craving, even at rest and in the absence of alcoholic stimuli. The intrusiveness of craving allows for an analogy with obsessive compulsive disorder (OCD). Interestingly, pathophysiology of OCD has also been related to increased structural connectivity between the OFC and the NAcc^25^. Our finding thus complements event-related fMRI studies demonstrating increased BOLD activations of the NAcc, and the OFC in response to alcoholic cues^36,44^. A previous event-related FC study using alcoholic cues investigated craving in a clinically heterogeneous patient group ranging from social drinkers to severe AUD^45^. Both positive (medial OFC-insula) and negative (lateral OFC-NAcc) correlations between FC during a cue reactivity task and craving were identified^45^. Differences in study design (resting-state vs. cue reactivity task) and clinical differences could account for the inconsistencies. Furthermore, craving may either be driven by reward (e.g., in social drinkers^67^) or by avoidance of negative affect (e.g., in severe AUD^42^). Thus, it is difficult to compare the neurobiology of craving between clinically diverse AUD-groups.

In line with our hypothesis, we found reduced volume of bilateral NAcc in AUD, thus replicating previous studies (e.g.,^68,69^). Furthermore, reduced FA of the left and reduced tract length of bilateral OFC-NAcc connections were found in AUD. Exploratory analysis showed an association (*r* = 0.387, *p* = 0.015, see Supplementary material Table 2) between the left NAcc volume and FA of the left OFC-NAcc segment. Thus, one may speculate whether alcohol-induced gray matter atrophy of the NAcc leads to reduced white matter microstructure in AUD. Given that the directionality (in terms of afferents or efferents) of fiber tracts cannot be determined based on tractography, it may also be possible that white matter microstructure alterations stem from cortical atrophy in the OFC, a common and well replicated finding in AUD^70^. Another explanation is that the toxic effects of alcohol damage both gray and white matter of the OFC-NAcc network^19,20,71^ or that white matter damage of the OFC-NAcc connection leads to retrograde atrophy of NAcc gray matter^72^. In either case, three complementary structural measures (FA and tract length of the OFC-NAcc connection and NAcc-volume) suggest atrophy of the OFC-NAcc network in AUD, most likely a consequence of alcohol-induced neurotoxicity^19,20,71^.

Contrary to our hypothesis, we did not find any group differences in resting-state OFC-NAcc FC between AUD and healthy controls. Previous studies demonstrated lower resting-state FC of the reward system in abstinent AUD patients than in HC^37^, a finding that is more pronounced in long-term than in short term AUD abstinence^38^. Given that our AUD group mainly consists of short-term abstinent patients this may have contributed to the absence of group differences regarding resting-state OFC-NAcc FC. However, previous findings are not entirely consistent. One longitudinal study demonstrated that contrary to the initial hypothesis, relapsers had lower FC between the PFC and the NAcc than abstainers^73^. Overall, divergent findings may stem from comparing results of anatomically adjacent but functionally complementary brain regions (e.g., different hotspots for wanting and liking within the NAcc shell or functional differences regarding the medial and the lateral OFC^74,75^. Furthermore, it is crucial to note that FC analyses are influenced by a series of factors such as AUD severity and duration, the presence of comorbid depressive symptoms, the duration of abstinence or the contextual situation regarding alcohol exposure, which may as well partially explain heterogeneous findings^38,42,45^.

Finally, this study has some limitations: First, our correlational study does not allow for conclusions regarding causalities or directionalities. Second, we use tractography to reconstruct individual projection pathways. However, it is impossible to determine the anatomical directionality of projection pathways. Furthermore, it is impossible to make statements on direct and indirect connections and on the type of neurotransmitters (e.g., dopaminergic or glutamatergic). Third, although AUD patients and healthy controls are well matched and analysis are controlled for age and gender, there remain differences (e.g., depressive symptoms, years of education). Given that education is strongly correlated with the group variable using education as covariate can reduce validity or sensitivity and is not advised^76^. Thus, we cannot statistically rule out that education may have had an influence on neurophysiological results. Fourth, our sample of AUD patients comprises almost exclusively patients with severe AUD. While this is a positive aspect allowing for a precise definition of the sample under investigation, it limits generalizability to patients with mild or moderate AUD.

To conclude, our findings in recently abstinent patients with severe AUD demonstrate that AUD is associated with gray and white matter structural alterations of the OFC-NAcc network, putatively a neurotoxic effect of alcohol. Functionally, this network plays a core role for craving as suggested by the identified correlation between craving and resting-state OFC-NAcc FC, which may also affect white matter microstructure of the corresponding OFC-NAcc connection pathway. Future studies should assess if those changes reverse with abstinence in the long term^24^. Furthermore, it is of clinical interest to assess the predictive value of such MRI-assessments for novel treatment approaches, such as neurofeedback, alcohol-inhibition training, or brain stimulation therapies^9–11^.

## Supplementary information

Supplementary material
