# Supplementary material for: The role of the orbitofrontal cortex and the nucleus accumbens for craving in alcohol use disorder
Source: Transl Psychiatry. 2021 May 4;11:267. doi: 10.1038/s41398-021-01384-w (PMC8097061; doi:10.1038/s41398-021-01384-w)

Supplementary table 1: Correlations for the AUD group between imaging modalities and OCDS scores

|  | NAcc (L) | NAcc (R) | FA (L) | FA (R) | Length (L) | Length (R) | FC (L) | FC (R) |
| --- | --- | --- | --- | --- | --- | --- | --- | --- |
| OCDS  (Thoughts) | R=0.228  P = 0.163 | R=0.008  P=0.960 | R=0.321*  P = 0.046 | R=0.058  P = 0.728 | R=0.081  P=0.624 | R=0.007  P=0.965 | R=0.477  P=0.002 | R=0.390  P=0.014 |
| OCDS  (Behavior) | R=0.174  P=0.289 | R=0.131  P=0.425 | R=0.174  P=0.289 | R=0.031  P=0.852 | R=-0.044  P=0.791 | R=-0.160  P=0.330 | R=0.245  P=0.132 | R=0.182  P=0.267 |
| OCDS  (Total) | R=0.199  P=0.224 | R=0.085  P=0.606 | R=0.262  P=0.107 | R=0.072  P=0.664 | R=0.036  P=0.827 | R=-0.083  P=0.615 | R=0.367*  P=0.022 | R=0.293  P=0.071 |

Supplementary table 2: Correlations between the imaging modalities for the AUD group

|  | NAcc (L) | NAcc (R) | FA (L) | FA (R) | Length (L) | Length (R) | FC (L) | FC (R) |
| --- | --- | --- | --- | --- | --- | --- | --- | --- |
| NAcc (L) |  | R=0.325*  P=0.044 | R=0.387*  P=0.015 | R=0.212  P=0.194 | R=-0.017  P=0.917 | R=0.145  P=0.379 | R=0.237  P=0.093 | R=0.014  P=0.934 |
| NAcc (R) |  |  | R=-0.088  P=0.592 | R=0.114  P=0.491 | R=0.116  P=0.483 | R=-0.028  P=0.868 | R=-0.044  P=0.789 | R=0.030  P=0.858 |
| FA (L) |  |  |  | R=0.655*  P<0.001 | R=0.316*  P=0.049 | R=0.504*  P=0.001 | R=0.028  P=0.865 | R=0.189  P=0.248 |
| FA (R) |  |  |  |  | R=0.399*  P=0.012 | R=0.548*  P<0.001 | R=-0.111  P=0.502 | R=0.084  P=0.611 |
| Length (L) |  |  |  |  |  | R=0.346*  P=0.031 | R=0.079  P=0.635 | R=0.416*  P=0.008 |
| Length (R) |  |  |  |  |  |  | R=0.091  P=0.582 | R=0.056  P=0.736 |

Supplementary figure 1: Whole-brain FC maps of NAcc within group and hemisphere.


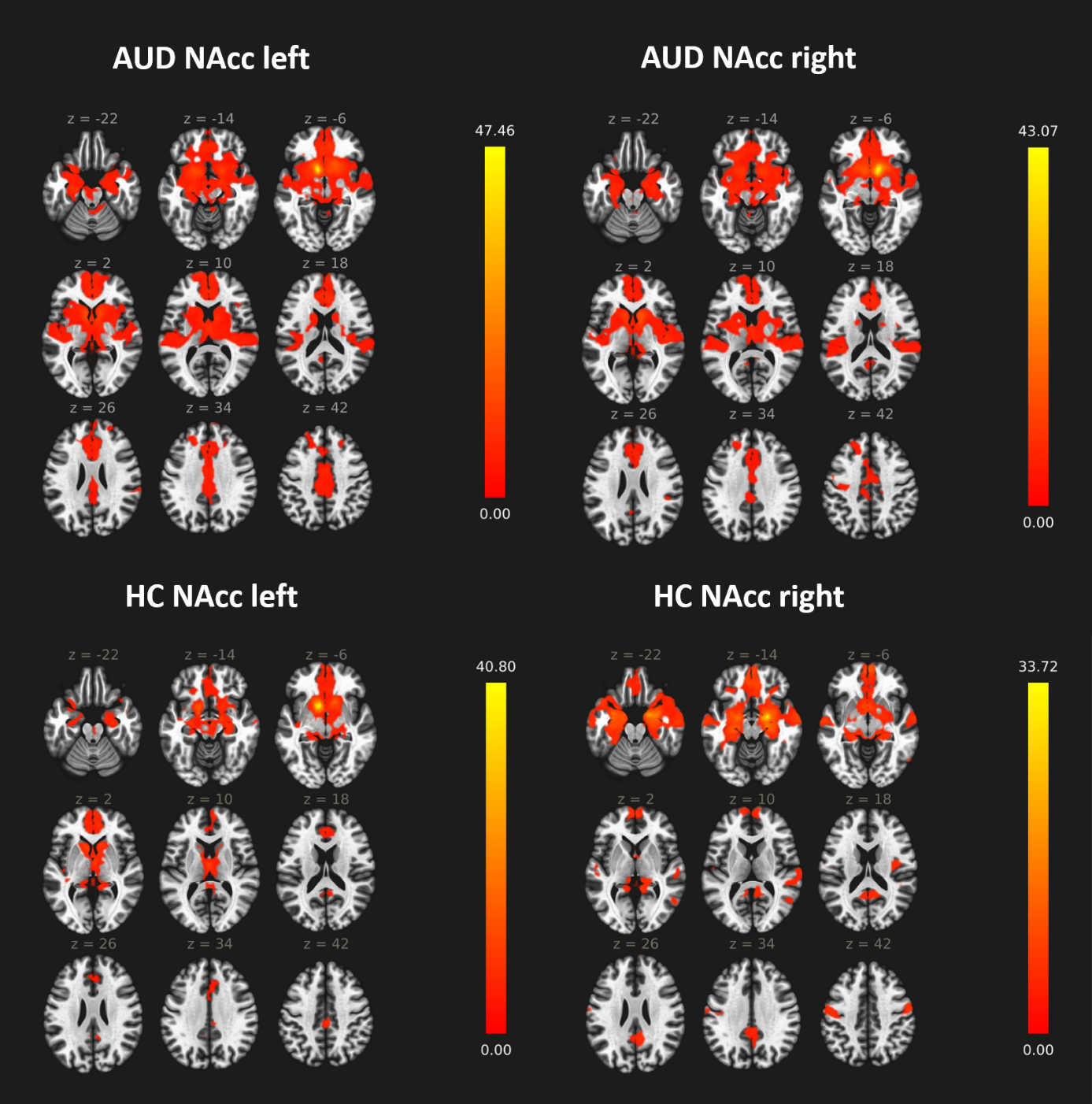

Supplement: Supplementary file 1 — Supplementary material [file 41398_2021_1384_MOESM1_ESM.docx]
